# The effect of an app-based dietary education on dietary intake and cardiometabolic risk markers in people with type 2 diabetes: results from a randomized controlled trial

**DOI:** 10.1186/s12937-024-01069-2

**Published:** 2025-01-04

**Authors:** Linnea Sjöblom, Essi Hantikainen, Anna Dahlgren, Ylva Trolle Lagerros, Stephanie E. Bonn

**Affiliations:** 1 Division of Clinical Epidemiology, Department of Medicine Solna, Karolinska Institutet, Eugeniahemmet T2:02, Stockholm, SE-171 76 Sweden; 2 Institute for Biomedicine, Eurac Research, Bolzano, 39100 Italy; 3 Department of Medicine Huddinge, Karolinska Institutet, Stockholm, Sweden

**Keywords:** Adults, Behavior change, Cardiovascular risk factors, Dietary change, Diabetes mellitus, Type 2, Diabetes management, mHealth, Smartphone application

## Abstract

**Background:**

mHealth, i.e. mobile-health, strategies may be used as a complement to regular care to support healthy dietary habits in primary care patients. We evaluated the effect of a 12-week smartphone-based dietary education on overall diet quality (primary outcome), and dietary intake and cardiometabolic risk markers (secondary outcomes) in people with type 2 diabetes.

**Methods:**

In this two-armed randomized clinical trial, people with type 2 diabetes were recruited within a primary care setting and randomized 1:1 to a smartphone-delivered dietary education for 12 weeks or a control group receiving regular care only. Dietary intake and cardiometabolic risk markers were measured at baseline and after 3 months. Diet was assessed using a 4-day dietary record and a food frequency questionnaire (FFQ). Overall diet quality was estimated with a Nordic Nutrition Recommendation (NNR) score and specific dietary intake was estimated for 13 food groups/nutrients. We used linear regression models to examine differences in change from baseline to the 3-month follow-up between the intervention and control group, adjusted for baseline values of each outcome variable.

**Results:**

The study included 129 participants (67 in the intervention group and 62 controls), of whom 61% were men. At baseline, mean age was 63.0 years and mean body mass index was 29.8 kg/m^2^. When analyzing dietary record data, we found no effect of the intervention on diet quality or intake, however, the control group had increased their score by 1.6 points (95%CI: -2.9, -0.26) compared to the intervention group. In the analyses of FFQ data, the intervention group had lowered their daily intake in grams of saturated (β = -4.1, 95%CI: -7.9, -0.2) and unsaturated (mono- and polyunsaturated) (β = -6.9, 95%CI: -13.5, -0.4) fat more than the control group. The intervention group also presented lower serum triglycerides levels than the controls (β = -0.33, 95%CI: -0.60, -0.05). No statistical differences were found in any other dietary variables or cardiometabolic risk markers.

**Conclusion:**

While we found no effect on overall diet quality, our findings suggest that a smartphone-based dietary education might impact dietary fat intake and corresponding cardiometabolic risk markers in people with type 2 diabetes. Our results should be considered hypothesis-generating and need to be confirmed in future studies.

**Trial registration:**

Registered at ClinicalTrials.gov (NCT03784612). Registered 24 December 2018.

**Supplementary Information:**

The online version contains supplementary material available at 10.1186/s12937-024-01069-2.

## Background

Diet plays a fundamental role in the management of type 2 diabetes to prevent its complications and to reduce the risk of cardiovascular disease [1]. High total cholesterol, low-density lipoprotein (LDL), and triglyceride levels, along with low high-density lipoprotein (HDL) levels, are associated with increased risk of cardiovascular disease [2, 3]. Since blood lipids are greatly impacted by our diet [4, 5], effective dietary strategies to lower serum lipid levels could complement medications. Further, improved dietary habits can reduce glycated hemoglobin (HbA1c) levels with an effect comparable to anti-diabetic medication for some [6].

Dietary recommendations for people with type 2 diabetes in most developed countries suggest a diet rich in vegetables, fruits, legumes, whole grains, nuts and seeds, and vegetable oils, while limiting refined grains, sugar-sweetened beverages, red and processed meats, and sodium [7–9]. Following a Mediterranean diet, which emphasizes the intake of fiber, legumes, and unsaturated fat, among other foods, enhances glycemic control [10], and decreases the risk of premature mortality [3]. A Nordic dietary pattern, similar to the Mediterranean diet but adapted to the Nordic region [11], has also been associated with a lower incidence of type 2 diabetes [12].

To target healthy eating habits and dietary intake among people with type 2 diabetes, mHealth solutions, i.e. mobile devices including smartphone applications (apps), have emerged as a new and promising way of delivering interventions. They are a cost-effective way to promote health [13] and have shown positive results on weight management and dietary intake. For example, a smartphone-based weight loss intervention with personalized recommendations and educational information demonstrated positive results on weight loss after 3 months among individuals with overweight or obesity [14]. App- or web-based self-monitoring has also been found to increase vegetable intake in individuals with overweight [15], reduce total energy and saturated fat intake [16], and significantly affect weight management [16, 17]. Furthermore, previous studies using various app solutions have shown to be effective for controlling weight and diabetes [18, 19]. Based on self-selected goals and health status, these apps provide recommendations for diet, exercise and lifestyle changes. Dietary interventions delivered by dietitians are effective in improving clinical markers, such as glycemic control and body weight in people with type 2 diabetes [20]. Both smartphone-based and face-to-face dietary education interventions lasting at least 3 months have been shown to be effective in lowering HbA1c levels [21]. However, data providing evidence of the efficacy of apps on actual dietary intake along with other cardiometabolic risk markers are lacking.

We aimed to evaluate the effect of a 12-week smartphone-based dietary education intervention compared to regular care on overall diet quality (primary outcome), and intake of fruits and vegetables, legumes, fish and seafood, red and processed meat, sugar-sweetened beverages, fiber, whole grains, carbohydrates, saturated and unsaturated fat, sucrose, sodium and total energy intake (secondary outcomes) among people with type 2 diabetes participating in a randomized clinical trial. We also examined the effect on cardiometabolic risk markers including body mass index (BMI), waist circumference, body fat percentage, HbA1c, serum lipid levels (i.e. triglycerides, and total-, HDL-, and LDL-cholesterol), and blood pressure (secondary outcomes).

## Material and methods

### Trial design

The Healthy eating using APP technologY (HAPPY) trial has been described in detail previously [22]. In brief, we performed a two-armed randomized clinical trial where participants were randomized 1:1 to an intervention group (smartphone-based dietary education during 12-weeks and regular care, i.e. care as usual at the primary care center) or a control group (regular care only). The active intervention spanned 12-weeks from baseline, during which time the intervention group received the dietary education. The control group was waitlisted at study start and offered the intervention after the 3-month follow-up. No changes to methods were made after trial commencement. This trial is reported in accordance with the CONSORT checklist (see Additional file 1).

### Study participants and recruitment

We recruited patients from five primary health care centers in Stockholm, Sweden, between January 2019 and August 2023. Data collection was temporarily paused in 2020–2021 due to the Covid-19 pandemic. The inclusion criteria were being diagnosed with type 2 diabetes according to international guidelines and treated within primary care, ≥ 18 years of age, able to read and understand Swedish, and having access to and being able to use a smartphone with a personal e-identification. No exclusion criteria were applied.

Patients were given brief information about the study from health care personnel at their regular health care visit to one of the participating primary care centers. Those who expressed interest to participate (*n* = 183) were thereafter contacted via telephone by study personnel and given additional information. In total, 133 patients agreed to participate and were scheduled for a physical baseline meeting with study personnel. Ahead of the meeting they received an e-mail with a link to the web-based baseline questionnaire. Study assessments were performed at baseline and at follow-up after 3 and 6 months. After 12 months, participants responded to a web-based questionnaire. Since the control group was given the dietary education at the 3-month follow-up, we are only able to address the intervention effect between baseline and 3 months. Therefore, in this study, we have only analyzed data from baseline and the 3-month follow-up to evaluate the intervention effect.

### Intervention

The smartphone-based intervention was designed to improve overall diet quality through a healthy eating education delivered via an app. The health belief model [23], stages of change model [24], and social cognitive theory [25] were used in the design of the app to encourage behavioral change. To support sustainable dietary changes, we used behavior change techniques such as goal-setting, educational information, self-monitoring and feedback [26]. The content focused on overall dietary habits and was based on existing Swedish national dietary recommendations and evidence-based guidelines [27].

**Fig. 1:**
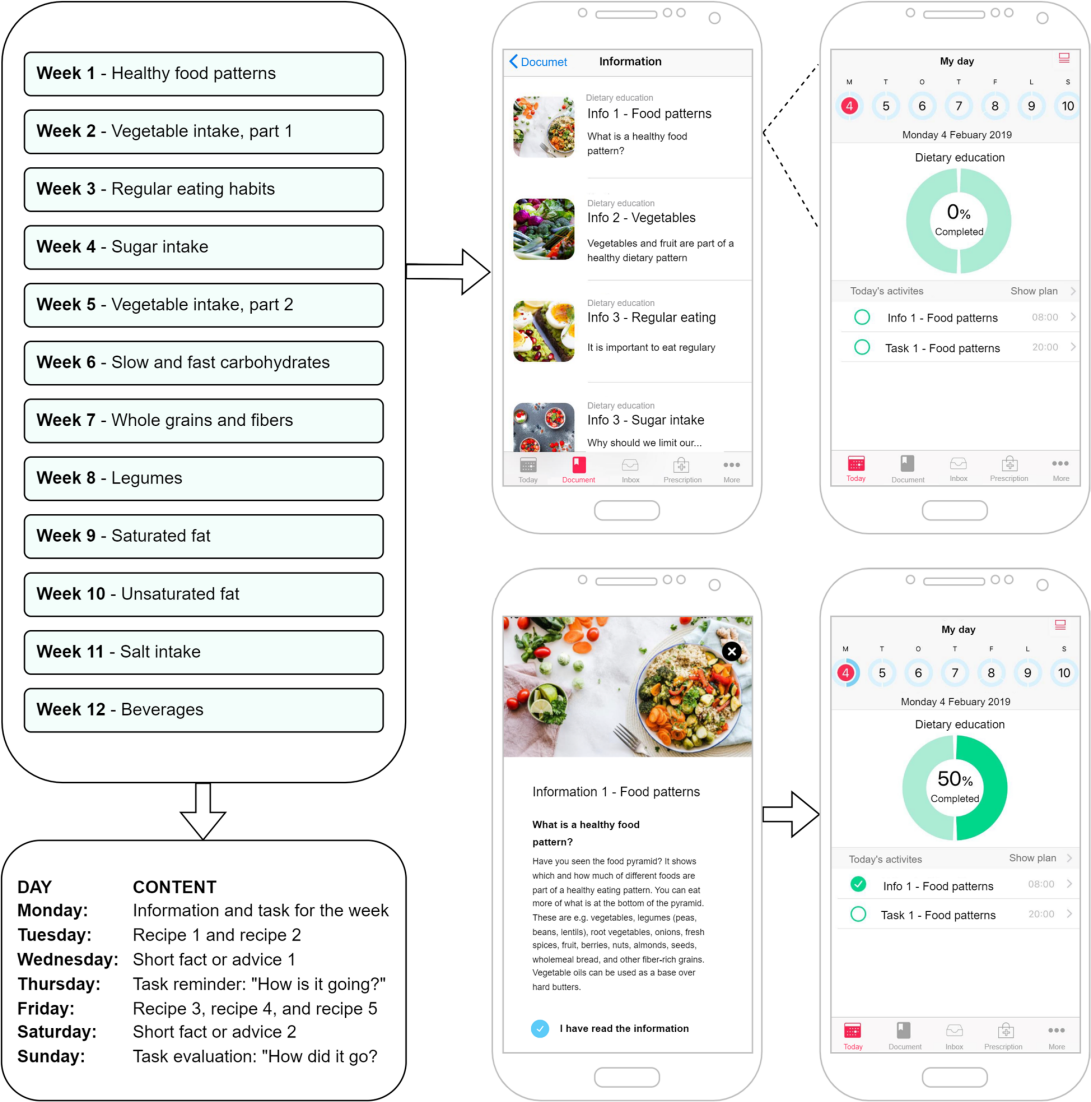
Overview of the different topics and content of the smartphone application and an example from the smartphone application from week 1 – Healthy food patterns. Before completing the activity, 0% is visualized, when the user has completed the activity “I have read the information”, the green circle is shown as 50% completed, and finally when the “task for the week” is completed the circle is shown as 100% of completed activities

Each week, a new topic was introduced according to a set schedule. The user received educational information, a weekly task to perform related to the topic, healthy recipes, practical advice, short fun facts, a reminder and an evaluation at the end of the week. The participants were encouraged to use the app daily, although this was not a requirement. The intervention was self-delivered and self-paced for personal progress. Automatic prompts/notifications were shown when a new activity was available in the app. Participants could contact study personnel if they detected any malfunctions in the app. Minor bug fixes were performed, but no content changes were made during the study. The app was developed specifically for this study and is no longer available. Participants downloaded the app together with study personnel and were registered to be able to log in to access the dietary education course. The app was free to download and came with no additional costs for the user. An overview of the different topics and examples of content in the app for week 1 (translated to English, original version in Swedish) are shown in Fig. 1.

### Dietary assessment

Dietary intake was primarily assessed using an estimated 4-day dietary record. Participants were asked to record information about their food and beverage intake in a given paper diary as precisely as possible over four consecutive days, including one weekend day. To obtain information on nutrient intakes, a nutritionist entered data from the diaries into the software Dietist Net [28]. This program is connected to the Swedish Food Composition Database from the Swedish National Food Agency [29]. If the portion size had not been recorded, standard portion sizes in Dietist Net were used. For unspecific food recordings, e.g. “fish or “cheese”, the most commonly consumed fish or cheese according to Swedish national data [30] was used. The food items included in the food groups in analysis and adjustments for the weight of the composite dishes can be found in the Supplementary material Table S1 (Additional file 2).

We additionally assessed dietary intake using a validated 95-item semi-quantitative self-administrated web-based food frequency questionnaire (FFQ) [31, 32]. In the FFQ, participants reported how often each item was consumed (e.g. once per day, week, or month). We obtained standard portion sizes from the Swedish Food Composition Database provided by the National Food Agency [29]. The consumption of foods and beverages was calculated as average daily intake in grams and then converted into daily intake in grams or micrograms for nutrients according to the Swedish Food Composition Database. If the FFQ was completed, but the participant had missing values for some variables in the FFQ, we assumed zero consumption of this food.

### Primary outcome – overall diet quality

Overall diet quality was addressed using a score defining adherence to the food-based dietary guidelines in the 2023 version of the Nordic Nutrition Recommendations (NNR) [33]. The NNR summarizes the available scientific evidence and provides nutrition recommendations and dietary guidelines for the Nordic and Baltic countries. The overall purpose is to promote health and prevent diet-related diseases such as type 2 diabetes and cardiovascular diseases [33]. We have previously used a nutrient-based NNR-score [34], but have now developed a food-based NNR-score to better reflect the food-based dietary guidelines.

The food based dietary guidelines in NNR include recommendations for increased consumption of vegetables, fruits and berries; cereals (indicated by the intake of whole grains); legumes; nuts and seeds; a moderate intake of total fish and seafood; white meat; milk and dairy and eggs; choosing vegetable oils over hard butters; and limiting intakes of red meat; sweets including sugar-sweetened beverages; and alcohol. We obtained intakes in g/day from both the dietary record and FFQ to estimate adherence to the food based dietary guidelines.

We based our NNR-score on ten different food groups from the guidelines: 1) vegetables, fruits, and berries; 2) whole grains (cereals); 3) pulses/legumes; 4) nuts and seeds; 5) total fish and seafood; 6) red meat; 7) milk and dairy; 8) vegetable oils; 9) sweets, including sugar-sweetened beverages (excluding salty snacks); and 10) alcohol. Potatoes, eggs, and white meat are included in the NNR, but were not included in the score because they are primarily included from an environmental perspective and lack specific recommended intake limits. We estimated a proportional score ranging from 0 to 3 points for each food group. Perfect adherence, when the intake was within the daily or weekly recommended intake range, was given 3 points. For intake of nuts and seeds, fish, milk and dairy products that have a recommended range of intake, we included a two-way proportion score for intakes above and below the recommended levels. The points for the ten food groups were summed into a total score, ranging from 0 to 30 points. Higher scores indicate greater adherence to the NNR. The recommended intakes for all the components included in the NNR-score are shown in the Supplementary material Table S2 (Additional file 2).

### Secondary outcomes – dietary intake

In addition to the overall diet quality score, we also obtained intakes from both the dietary record and FFQ for outcomes corresponding to topics covered in the dietary education. These were total fruit and vegetables, including root vegetables and berries (covered during weeks 1, 2 and 5); total energy (kcal/day) (week 3); sucrose (week 4); carbohydrates (week 6); fiber and whole grains (weeks 6 and 7); legumes (week 8); red and processed meat (week 9); saturated fat (week 9); unsaturated fat (weeks 10); total fish and seafood (weeks 1 and 10); salt expressed as sodium (week 11); and sugar-sweetened beverages, including fruit juice (week 12). All consumption units were expressed as g/day, except sodium, which was expressed as mg/day.

### Secondary outcomes – cardiometabolic risk markers

We measured anthropometric variables including weight to the nearest 0.1 kg with light clothing without shoes using an electronic scale, and height to the nearest 0.5 cm without shoes using a measuring stick. BMI was calculated based on measured weight and height (kg/m^2^) and participants were categorized as normal weight (< 25.0 kg/m^2^), overweight (25.0–29.9 kg/m^2^), or having obesity (≥ 30.0 kg/m^2^) [35]. Self-reported BMI from the baseline questionnaire was used for individuals who could not attend the baseline meeting (*n* = 4). Waist circumference was measured about two cm above the umbilicus to the nearest cm. We created two categories corresponding to low risk (< 88 cm for women and < 102 cm for men) and high risk (≥ 88 cm for women and ≥ 102 cm for men) of disease according to the World Health Organization (WHO) cut-off values [35]. Body composition, including body fat percentage, was measured using a digital body composition scale (Tanita, Model BC-418) [36].

Fasting blood sampling of HbA1c (mmol/mol) [37] and serum lipid levels (mmol/L) [38] including triglycerides (mmol/L) [39], total cholesterol (mmol/L), LDL-cholesterol [39], and HDL-cholesterol (mmol/L) [40], were collected at baseline and after 3- and 6-months. Blood samples were analyzed at a lab affiliated with Karolinska University Hospital. Full details of the biomarker measurement procedure are described in the protocol [22]. Briefly, triglycerides were measured using the enzymatic method and the Friedewald equation was used to calculate the concentration of LDL cholesterol [40]. We further calculated the levels of non-HDL cholesterol (non-HLD = total cholesterol – HDL-cholesterol). In the first 35 study participants, blood pressure (systolic and diastolic) was measured manually at baseline and 3-month follow-up. Thereafter, blood pressure was measured using the automatic electronic monitor OMRON M7.

### Background characteristics

Age, sex (female, male), education level (≤ 12 years, > 12 years), smoking status (never/former, current), diabetes duration (≤ 5 years, > 5 years) and medication for hypertension, hyperlipidemia, and diabetes were self-reported in the baseline questionnaire. Physical activity (min/week) was estimated with two questions asking about the time spent on exercise, such as running, training sessions or ball games during a typical week and daily physical activity, such as walking, cycling or gardening, used in routine care for people with type 2 diabetes [41]. We created a dichotomous variable (< 150 min/week, ≥ 150 min/week) based on the recommendations from the WHO for physical activity of at least moderate intensity [42].

### Power calculation

The power calculation was based on HbA1c as the outcome variable, since this is the key marker for monitoring type 2 diabetes. Furthermore, it is an objective measure compared to dietary intake, which can be defined in different ways. A sample size of 168 participants (84 per group) was calculated to be needed for the detection of a 4 mmol/mol change in HbA1c (80% power, 5% significance level) [22]. Baseline data collection ended in August 2023 before 168 participants had been included because the smartphone application was no longer compatible with the upgrades of iOS and Android.

### Randomization and blinding

A random allocation sequence was computer-generated by SEB in Stata, version 14.0 (Stata Corporation, College Station, TX, USA). Randomization 1:1 was performed by study personnel (LS and AD) separately for women and men in blocks of four within each primary care center. Each participant was consecutively assigned in the allocation sequence list without knowing their specific group allocation until the baseline measurements were completed. The laboratory personnel who analyzed the blood samples were blinded, but neither the participants nor the study personnel who performed the data analysis were blinded due to the nature of the intervention.

### Statistical analysis

Descriptive statistics are presented as numbers with percentages (%) and means with standard deviations (SD) for categorical and continuous variables, respectively. Differences in baseline characteristics between the intervention and control group were assessed using Student’s t-test and chi-square test. Adherence to the smartphone app was assessed by the proportion (%) of activities completed during the intervention period.

Analysis of the intervention effect were performed using an intention-to-treat approach. We fitted linear regression models to analyze the difference in change in outcomes from baseline to the 3-month follow-up between the intervention and control group, where each model was additionally adjusted for baseline values of the specific outcome variable to account for differences at baseline [43]. The mean difference in change between groups (represented by the β-coefficient) with 95% confidence intervals (CI) was calculated. Normality of the resulting residuals of each model was visually evaluated using histograms with normal curve overlay and Q-Q (quantile–quantile) plots. Since normality was violated for intake of sugar-sweetened beverages and legumes, both from the dietary record and the FFQ, we instead ran rank-based ANCOVA tests separately for these variables. The resulting *p*-values for the group differences are presented. Participants with complete dietary data from the dietary records at baseline and at the 3-month follow-up were included in the primary analyses of the intervention effect on dietary intake. We also performed additional analyses using FFQ data, which had less missing data than the dietary records. A sensitivity analysis comparing baseline data among those who dropped out or had missing outcome data, with baseline data among those who continued within the study was performed using student t-test and chi^2^ test to evaluate any differences. Statistical tests were two-sided, and the statistical significance level was set to *p* < 0.05. We conducted the statistical analysis using Stata version 17.0 (Stata Corporation, College Station, TX, USA).

## Results

**Fig. 2:**
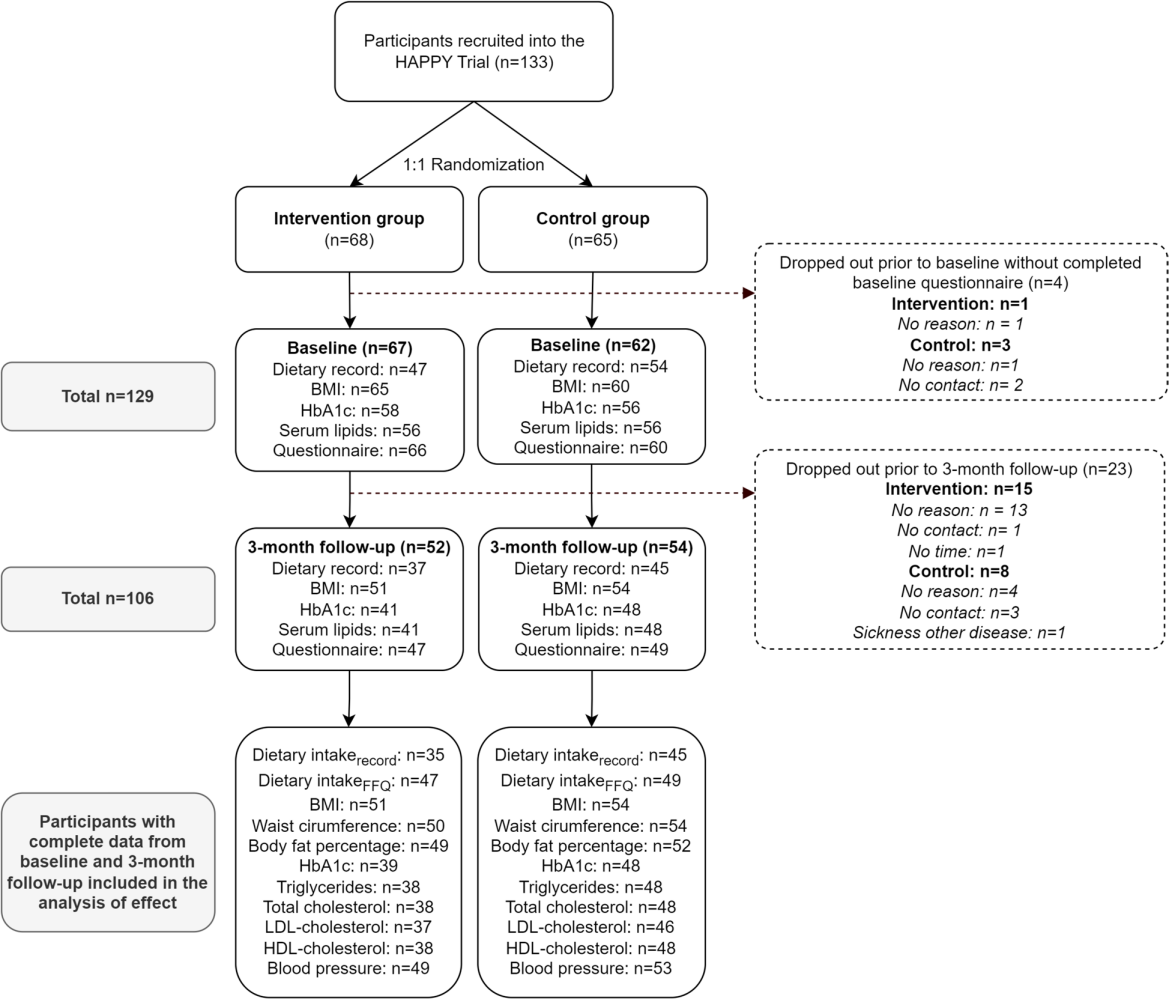
Flow diagram of participants in the HAPPY trial

A total of 133 participants met the inclusion criteria, agreed to participate, and were randomized. Four individuals dropped-out before providing any baseline data, i.e. before being made aware of their group allocation, leaving 129 participants to be included in the study. A total of 101 participants (78%) completed the baseline dietary record and 125 (97%) completed the baseline FFQ. Fewer participants in the intervention group than in the control group had complete data from the dietary record at both baseline and at the 3-month follow-up (*p* = 0.02). No differences in baseline characteristics were observed between participants with complete data from both time points and those with missing data or those who dropped out, neither among all nor within the intervention and control groups. A flowchart of participants in the intervention and control group, respectively, with complete data from the different study assessments are shown in Fig. 2.

Baseline characteristics of the 129 participants (61.2% men) randomized to the intervention group (*n* = 67) or control group (*n* = 62) are presented in Table 1. Mean age was 63.0 (SD 10.1) years, mean BMI 29.8 (SD 5.0) kg/m^2^ and average HbA1c 49.6 (SD 10.4) mmol/mol. Most of the participants met physical activity recommendations (77.8%), were non-smokers (95.0%) and had over 12 years of education (59.7%). There were no statistically significant differences in the baseline characteristics between the study groups. In the intervention group, adherence to the intervention was good. On average, 71.3% (SD: 39.4) of all 136 app activities were completed.

**Table 1:** Baseline characteristics of all participants by intervention and control group

	**All (*n* = 129)**			**Intervention (*n* = 67)**			**Control (*n* = 62)**			
**Characteristics**	n	mean	(SD)	n	mean	(SD)	n	mean	(SD)	*p*-value^*^
Age (years)	129	63.0	(10.1)	67	63.3	(9.7)	62	62.7	(10.7)	0.77
Total energy intake (kcal/day)	101	1911	(484)	47	1946	(522)	54	1881	(451)	0.50
Physical activity (min/week)	126	284	(153)	66	274	(159)	60	296	(147)	0.44
		n	(%)		n	(%)		n	(%)	
**Sex**										0.71
Female		50	(38.8)		27	(40.3)		23	(37.1)	
Male		79	(61.2)		40	(59.7)		39	(62.9)	
**Education**^**a**^										0.51
≤ 12 years		50	(40.3)		28	(43.1)		22	(37.3)	
> 12 years		74	(59.7)		37	(56.9)		37	(62.7)	
**Current smoker (yes)**^**b**^		5	(4.0)		3	(4.5)		2	(3.4)	0.74
**Physical activity**^**c**^										0.32
< 150 min/week		28	(22.2)		17	(25.8)		11	(18.3)	
≥ 150 min/week		98	(77.8)		49	(74.2)		49	(81.7)	
**BMI categories**^**d**^										0.45
Normal weight, < 25.0 kg/m^2^		21	(16.3)		12	(17.9)		9	(14.5)	
Overweight, 25.0–29.9 kg/m^2^		53	(41.1)		24	(35.8)		29	(46.8)	
Obese, ≥ 30.0 kg/m^2^		55	(42.6)		31	(46.3)		24	(38.7)	
**Waist circumference**^**b**^										0.95
Low		33	(26.4)		17	(26.2)		16	(26.7)	
High		92	(73.6)		48	(73.9)		44	(73.3)	
**Diabetes duration**^**e**^										0.74
≤ 5 years		73	(60.8)		38	(62.3)		35	(59.3)	
> 5 years		47	(39.2)		23	(37.7)		24	(40.7)	
**Medical use (yes)**^**c**^		79	(62.7)		41	(62.1)		38	(63.3)	0.89
**Type of medical use (yes)**^**c**^										
Blood pressure medication		83	(65.9)		45	(68.2)		38	(63.3)	0.57
Insulin		21	(16.7)		9	(13.6)		12	(20.0)	0.34
Oral medication for diabetes, e.g. Metformin		95	(75.4)		47	(71.2)		48	(80.0)	0.25
Medication for blood lipids		79	62.7		41	(62.1)		38	(63.3)	0.89

*Abbreviations*: *SD* standard deviation, *BMI* body mass index, *LDL* low-density lipoprotein, *HDL* high-density lipoprotein

^*^Differences between groups using two-tailed t-test (continuous variables) and chi^2^ test (categorical variables). Statistical significance is defined as *p* < 0.05

^a^Missing data *n* = 5

^b^Missing data *n* = 4

^c^Missing data *n* = 3

^d^self-reported bmi *n* = 4

^e^Missing data *n* = 9

Table 2 shows results of adherence to the NNR-score and dietary intake based on the dietary record, FFQ and levels of cardiometabolic risk markers at baseline and at the 3-month follow-up. In the intervention group, the mean NNR-score was 10.9 at baseline and 10.6 at follow-up, while the mean score was 10.8 at baseline and 12.1 at follow-up in the control group. When using FFQ data the mean NNR-scores were higher compared to record data. The mean scores from the FFQ were 13.4 (SD 3.4) at baseline and 13.9 (SD 3.4) at follow-up in the intervention group and 13.6 (SD 3.3) and 14.0 (SD 3.6) at corresponding time points in the control group. There were no statistically significant differences in NNR-score or other variables of dietary intake from the dietary record or the FFQ between the study groups at baseline. Similarly, no statistically significant differences in cardiometabolic risk markers were seen between the intervention and control group at baseline.

**Table 2:** Mean dietary intakes and Nordic Nutrition Recommendations 2023 (NNR_2023_) score from the dietary record and food frequency questionnaire (FFQ), and mean cardiometabolic risk markers between baseline and 3-month follow-up among participants with complete data from both time points, stratified by intervention and control group

	**Intervention (*n* = 35)**				**Control (*n* = 45)**				Between groups
	Baseline		3-month		Baseline		3-month		at baseline
	mean	(SD)	Mean	(SD)	mean	(SD)	mean	(SD)	*p*-value^*^
NNR-score (0–30 points) (dietary record)	10.9	(3.6)	10.6	(2.9)	10.8	(3.5)	12.1	(3.6)	0.91
**Dietary variables (dietary record)**									
Fruit and vegetables (g/day)	288	(162)	284	(162)	252	(157)	306	(190)	0.33
Legumes/pulses (g/day)	15	(32)	15	(32)	8	(16)	11	(22)	0.20
Total fish and seafood (g/day)	47	(40)	58	(51)	47	(41)	41	(34)	0.98
Red and processed meat (g/day)	86	(58)	92	(58)	98	(66)	95	(64)	0.41
Sugar-sweetened beverages (g/day)	46	(116)	29	(58)	48	(75)	23	(59)	0.94
Fiber (g/day)	22	(9)	20	(7)	23	(12)	22	(10)	0.94
Whole grains (g/day)	39	(39)	33	(32)	34	(25)	35	(20)	0.51
Carbohydrates (g/day)	177	(58)	160	(51)	177	(61)	170	(48)	0.99
Saturated fat (g/day)	32	(12)	27	(10)	32	(14)	30	(9)	0.91
Unsaturated fat (g/day)	41	(13)	41	(20)	41	(17)	42	(13)	0.93
Sodium (mg/day)	2908	(854)	2700	(793)	3055	(1004)	3125	(1005)	0.49
Sucrose (g/day)	14.0	(11.6)	11.0	(8.7)	15.0	(8.9)	11.2	(8.8)	0.62
Total energy (kcal/day)	1922	(496)	1744	(505)	1928	(455)	1827	(312)	0.95
NNR-score (0–30 points) (FFQ)	13.4	(3.4)	13.9	(3.4)	13.6	(3.3)	14.0	(3.6)	0.88
**Dietary variables (FFQ)**									
Fruit and vegetables (g/day)	353	(236)	368	(195)	372	(249)	420	(305)	0.69
Legumes/pulses (g/day)	40	(41)	48	(48)	34	(36)	53	(61)	0.51
Total fish and seafood (g/day)	51	(30)	52	(31)	45	(28)	44	(26)	0.27
Red and processed meat (g/day)	80	(42)	75	(39)	93	(78)	84	(56)	0.32
Sugar-sweetened beverages (g/day)	13	(67)	22	(78)	4	(30)	13	(66)	0.39
Fiber (g/day)	28	(14)	29	(12)	28	(10)	32	(13)	0.88
Whole grains (g/day)	65	(31)	65	(32)	63	(36)	68	(35)	0.80
Carbohydrates (g/day)	208	(80)	206	(75)	207	(69)	223	(74)	0.95
Saturated fat (g/day)	32	(11)	31	(11)	32	(13)	35	(15)	0.86
Unsaturated fat (g/day)	50	(21)	48	(18)	47	(17)	52	(25)	0.44
Sodium (mg/day)	2684	(886)	2683	(808)	2655	(820)	2821	(802)	0.87
Sucrose (g/day)	32	(16)	31	(14)	30	(13)	34	(17)	0.55
	**Intervention (*n* = 51)**				**Control (*n* = 54)**				
**Cardiometabolic risk markers**									
BMI (kg/m^2^)	29.8	(5.2)	29.5	(5.2)	29.7	(4.2)	29.4	(4.2)	0.94
Waist circumference (cm)^a^	105.4	(13.8)	103.2	(14.0)	105.7	(12.2)	104.2	(11.7)	0.90
Body fat (%)^b^	32.0	(9.4)	31.7	(9.3)	32.1	(8.9)	32.4	(8.6)	0.96
HbA1c (mmol/mol)^c^	49.4	(10.1)	48.2	(9.8)	50.3	(10.1)	50.4	(12.7)	0.66
Triglycerides (mmol/L)^d^	1.5	(0.9)	1.3	(0.7)	1.6	(0.8)	1.7	(1.0)	0.49
Total cholesterol (mmol/L)^d^	4.0	(0.9)	4.0	(0.8)	4.2	(1.2)	4.3	(1.3)	0.43
LDL-cholesterol (mmol/L)^d,e^	2.2	(0.7)	2.1	(0.6)	2.3	(1.0)	2.3	(1.0)	0.48
HDL-cholesterol (mmol/L)^d^	1.3	(0.4)	1.3	(0.4)	1.2	(0.3)	1.2	(0.4)	0.10
Non-HDL-cholesterol^d^	2.7	(0.9)	2.7	(0.8)	3.0	(1.2)	3.1	(1.3)	0.19
Blood pressure (mmHg)^f^									
*Systolic*	135	(15)	132	(16)	135	(15)	131	(13)	0.90
*Diastolic*	85	(10)	84	(11)	83	(13)	80	(10)	0.28

*Abbreviations*: *SD* standard deviation, *FFQ* food frequency questionnaire, *BMI* body mass index, *LDL* low-density lipoprotein, *HDL* high-density lipoprotein

^*^Differences between groups at baseline with complete data from both time points using two-tailed t-test

^a^missing *n* = 1

^b^missing *n* = 4

^c^missing *n* = 18

^d^missing *n* = 19

^e^additional missing *n* = 3 due to restrictions in the Friedewald equation to calculate LDL cholesterol with high triglyceride levels

^f^missing *n* = 3

### Effectiveness of intervention

Results of the intervention effects on dietary intake estimated from the dietary records, FFQ and for clinical markers assessed by the linear regression models are presented in Table 3. We found a significant lower reduction of the NNR-score in the intervention group compared to the control group (β = -1.6, 95% CI: -2.9 to -0.26). We found no statistically significant effect of the intervention on any of the other dietary variables assessed with the dietary record. Estimates remained similar in the analysis using FFQ data, but became statistically significant for some variables of intake. Greater reductions in saturated fat intake (β = -4.1, 95% CI: -7.9 to -0.18) and unsaturated (mono- and polyunsaturated) fat intake (β = -6.9, 95% CI: -13.5 to -0.4) were observed in the intervention group as compared to the control group. In the analysis of cardiometabolic risk markers, the intervention group had lowered their serum triglyceride levels more than the control group (β = -0.33, 95% CI: -0.60 to -0.05), but no other statistically significant differences between groups were seen.

**Table 3:** Mean values for differences between baseline and 3-month follow-up in the intervention and control group and the intervention effect presented as difference in changes (β-coefficients and 95% confidence intervals) between groups in the HAPPY trial

	**Intervention (*n* = 35)**		**Control (*n* = 45)**			
	Difference		Difference		Model estimates^a^	
	mean	(SD)	mean	(SD)	β	(95% CI)
NNR-score (0–30 points) (dietary record)	-0.3	(3.6)	1.3	(3.3)	-1.6	(-2.9 to -0.3)
**Dietary variables (dietary record)**						
Fruit and vegetables (g/day)	-3.9	(164.3)	54.1	(150.9)	-45.4	(-111.9 to 21.1)
Legumes/pulses (g/day)	0.0	(35.9)	3.1	(23.9)	-^b^	
Total fish and seafood (g/day)	10.4	(54.7)	-6.3	(44.9)	17.1	(-1.1 to 35.3)
Red and processed meat (g/day)	5.9	(59.0)	-3.0	(56.5)	3.7	(-19.2 to 26.6)
Sugar-sweetened beverages and juice (g/day)	-16.8	(78.9)	-25.1	(62.7)	-^b^	
Fiber (g/day)	-2.9	(8.2)	-0.4	(7.2)	-2.6	(-5.4 to 0.3)
Whole grains (g/day)	-6.0	(26.7)	1.1	(22.5)	-4.8	(-13.5 to 3.9)
Carbohydrates (g/day)	-17.4	(43.0)	-7.4	(42.7)	-9.9	(-25.6 to 5.8)
Saturated fat (g/day)	-4.9	(9.7)	-2.2	(10.6)	-2.5	(-6.0 to 1.0)
Unsaturated fat (g/day)	-0.2	(20.0)	0.2	(16.3)	-0.6	(-7.5 to 6.4)
Sodium (mg/day)	-206.8	(831.0)	70.2	(1110.3)	-363.4	(-740.3 to 13.5)
Sucrose (g/day)	-3.2	(12.2)	-3.9	(12.4)	-0.3	(-4.2 to 3.6)
Total energy (kcal/day)	-177.6	(409.9)	-101.7	(402.0)	-79.2	(-230.0 to 71.5)
NNR-score (0–30 points) (FFQ)	0.4	(2.7)	0.4	(3.4)	-0.02	(-1.2 to 1.1)
**Dietary variables (FFQ)**						
Fruit and vegetables (g/day)	15.0	(150.9)	48.2	(232.8)	-38.4	(-114.2 to 37.4)
Legumes/pulses (g/day)	8.9	(42.6)	18.7	(46.9)	-^b^	
Total fish and seafood (g/day)	0.1	(30.0)	-1.4	(22.8)	4.3	(-5.2 to 13.9)
Red and processed meat (g/day)	-4.7	(36.7)	-9.3	(65.4)	-2.8	(-19.1 to 13.6)
Sugar-sweetened beverages and juice (g/day)	8.8	(60.3)	8.4	(59.1)	-^b^	
Fiber (g/day)	0.9	(11.1)	3.8	(9.9)	-2.7	(-6.6 to 1.1)
Whole grains (g/day)	-0.0	(29.2)	4.5	(30.2)	-3.8	(-14.6 to 7.0)
Carbohydrates (g/day)	-1.6	(56.8)	16.6	(62.9)	-17.9	(-40.2 to 4.5)
Saturated fat (g/day)	-2.8	(23.4)	3.2	(10.8)	-4.1	(-7.9 to -0.18)
Unsaturated fat (g/day)	-1.8	(13.9)	5.7	(18.6)	-6.9	(-13.5 to -0.4)
Sodium (mg/day)	-1.8	(755.4)	165.7	(690.7)	-155.5	(-412.7 to 101.8)
Sucrose (g/day)	-0.6	(12.1)	4.0	(15.7)	-4.0	(-9.3 to 1.3)
Total energy (kcal/day)	-37.1	(485.5)	172.9	(595.0)	-195.7	(-401.7 to 10.3)
	**Intervention (*n* = 51)**		**Control (*n* = 54)**			
**Cardiometabolic risk markers**						
BMI (kg/m^2^)	-0.3	(0.7)	-0.3	(0.7)	0.04	(-0.23 to 0.32)
Waist circumference (cm)^c^	-2.2	(4.0)	-1.6	(3.3)	-0.69	(-2.09 to 0.71)
Body fat (%)^d^	-0.3	(2.7)	0.2	(2.2)	-0.56	(-1.51 to 0.40)
HbA1c, Glycated hemoglobin (mmol/mol)^e^	-1.1	(7.1)	0.1	(9.5)	-1.43	(-4.99 to 2.14)
Triglycerides (mmol/L)^f^	-0.1	(0.6)	0.2	(0.8)	-0.33	(-0.60 to -0.05)
Total cholesterol (mmol/L)^f^	-0.1	(0.8)	0.1	(0.9)	-0.22	(-0.55 to 0.11)
LDL-cholesterol (mmol/L)^g^	-0.1	(0.4)	-0.1	(0.8)	-0.03	(-0.29 to 0.24)
HDL-cholesterol (mmol/L)^f^	0.0	(0.2)	0.1	(0.2)	-0.02	(-0.10 to 0.05)
Non-HDL-cholesterol (mmol/L)^h^	-0.1	(0.7)	0.0	(0.8)	-0.22	(-0.54 to 0.08)
Blood pressure (mmHg)^g^						
*Systolic*	-3.8	(13.8)	-3.8	(13.7)	0.26	(-4.39 to 4.91)
*Diastolic*	-1.5	(9.8)	-3.4	(10.5)	3.07	(-0.31 to 6.45)

*Abbreviations*: *BMI* Body Mass Index, *FFQ* food frequency questionnaire, *LDL* low-density lipoprotein, *HDL* high-density lipoprotein, *HAPPY* Healthy Eating using APP technology, *NNR* Nordic Nutrition Recommendations

^a^Results from the linear regression model adjusted for baseline values for the mean changes between groups

^b^Rank-based ANCOVA was used because of violation of the normality assumption, all *p-*values > 0.05

^c^missing *n* = 1

^d^missing *n* = 4

^e^missing *n* = 18

^f^missing *n* = 19

^g^missing *n* = 22

^h^missing *n* = 3

## Discussion

In this randomized controlled trial evaluating the effectiveness of a 12-week smartphone-based dietary education intervention among people with type 2 diabetes, we found no intervention effect on overall diet quality i.e. adherence to the NNR guidelines. A statistically significant intervention effect was shown on dietary intake of saturated and unsaturated fat in analyses of FFQ data, and the intervention group had significantly improved serum triglyceride levels over the control group. No other measure of dietary intake, or any other cardiometabolic risk marker, were statistically significant between the groups.

### Results in context

In contrast to our results of no effect on overall diet quality, increased adherence to the Mediterranean diet and another diet quality index was observed after 3 months of follow-up in a multifactorial smartphone app intervention targeting individuals with type 2 diabetes [44]. In addition to access to a smartphone app, the intervention also included a food workshop with groups of 10 participants that had theoretical and practical workshops aiming to support adherence to the Mediterranean diet and weekly group walks for five weeks. Another mHealth intervention using text-messaging found no statistically significant effect of the intervention on adherence to the Mediterranean diet [45]. Surprisingly, in our study, the control group had increased their NNR-score more than the intervention group. There may be several possible explanations for this. Enrolling into a dietary study might be motivation enough to change dietary habits [46], although this may not explain the lack of change in the intervention group. Although participants in the control group did not receive the intervention, they were free to search for information and/or support on healthy eating habits outside of the intervention, which may have included more support than an app alone.

One explanation to our results of an effect of the intervention on fat intake, but not overall diet quality, could be that interventions targeting specific behaviors potentially are more successful in creating a detectable and clinically relevant behavior change within that specific area, rather than trying to achieve changes in a broader and less defined context. Behavioral changes are challenging and concrete goals are therefore important. Mummah et al. [15] reported a significant increase in vegetable consumption among adults with obesity after 8 weeks using a theory-based smartphone app specifically focusing on vegetable consumption. Like our study, this intervention included features such as goal setting, self-monitoring, and feedback and was designed according to the social cognitive theory [25]. However, Mummah et al. focused only on vegetable intake, whereas we focused on overall dietary habits. It is possible that to achieve a measurable change in dietary intake, interventions should focus on intake of selected food items or need a longer time frame in order for new behaviors to emerge.

Our results on a potential intervention effect on fat intake are in line with several previous interventions [47–49]. A randomized controlled trial in adults with obesity revealed that an intervention comprising tailored feedback delivered via smartphones during 6 months resulted in reduced intake of saturated fat and total energy [47]. Additionally, another mHealth intervention reported lower intake of cholesterol and full-fat dairy products following an intervention with several dietary intake records during a 3-month period [48]. Lim et al. [49] evaluated a 6-month long smartphone-based lifestyle intervention including both diet and physical activity among individuals with type 2 diabetes in an Asian population and reported positive effects on the intake of total- and saturated fat, carbohydrates, sugar, and energy in the intervention group compared to the control group. In our dietary education program, weeks 9 and 10 focus on fat intake and recommendations to reduce saturated fat and replace these products with unsaturated fat. Participants were also encouraged to choose low-fat products. It is possible that we are detecting an instant effect on fat intake. Long-term effects should be evaluated, however, since our control group received the intervention at 3 months, this was not possible to address using our data. Similar to our findings, in a meta-analysis [3], a low-fat diet in combination with physical activity, compared to a regular diet, was also shown to lower the serum levels of triglycerides (-0.28; 95% CI: -0.45 to -0.10 mmol/L) after 6 months in individuals with type 2 diabetes based on data from three previous randomized controlled trials [50–52]. Lower levels of serum triglycerides could be a result of a decreased intake of dietary fat, as well as a lower intake of carbohydrates. Both play a role in the biological mechanism linking dietary intake and serum lipid levels [53].

Previous mHealth interventions targeted towards people with type 2 diabetes have often focused on the effect on glycemic control with HbA1c as a primary outcome [54–56], while less focus has been given to dietary changes [57]. In a systematic review and meta-analysis, Kim et al. [21] found that dietary education interventions provided for at least 3 months resulted in lower HbA1c among people with type 2 diabetes. We found no effect on HbA1c in our study. In a smartphone-based intervention among individuals with type 2 diabetes, the intervention group recorded their dietary intake in an app, used a glucometer and received real-time advice and feedback on their glucose levels [58]. As a result, the intervention group significantly lowered their HbA1c levels after 6 months compared to the control group. The frequent use of a glucometer could explain why participants became extra motivated to make changes that reduced their HbA1c levels. We did not include glucose monitoring in our intervention as we wanted to evaluate the effect of an educational intervention alone, without the additional impact of glucose monitoring.

In contrast to our null-findings on HbA1c, BMI, and blood pressure, Lim et al. [49] found that their smartphone-based lifestyle intervention including both diet and physical activity was more effective in reducing HbA1c, weight and diastolic blood pressure compared to regular care. However, since their intervention focused on both diet and physical activity, it is difficult to distinguish which part of the intervention affected the cardiometabolic risk markers. Furthermore, Alonso-Domínguez et al. [44] found that their multifactorial intervention in people with type 2 diabetes led to lower waist circumference, BMI, and postprandial glucose in the intervention group compared to the control group at 3 months, but not at 12 months. However, like our study, no intervention effect was seen for HbA1c, total cholesterol, LDL-cholesterol, HDL-cholesterol or blood pressure. One explanation for not observing an effect on either BMI or waist circumference in our study could be that we did not focus on weight loss.

### Strengths and limitations

The randomized study design and the high adherence to the app, with over 70% completing all activities, are strengths of our study. Another advantage is that we recruited participants within primary care, which may increase the generalizability of our results. However, the Swedish language in the app may have led to a selection of participants, excluding recently arrived immigrants. Nevertheless, the BMI in our study population reflects average patients with type 2 diabetes in primary care in Sweden [59]. We have similar proportions of overweight or obese individuals in our study population (83.4%) as in primary care (83.7%). However, our participants were younger (63.0 years vs. 68.7 years) and had a slightly better HbA1c (49.6 mmol/mol vs. 52.0 mmol/mol) than people with type 2 diabetes in Sweden.

Another strength of our study is the inclusion of both women and men. The proportion of men in our study (61.2%) is comparable to the proportion of men with type 2 diabetes within primary care in Sweden (58.1%) [59]. Men, especially older men, have been shown to be difficult to recruit in lifestyle interventions compared to women [60]. Moreover, we assessed dietary intake with a dietary record, which is considered a gold standard for assessing food intake [61]. In addition, we used a validated FFQ [31, 32]. Nevertheless, it should be acknowledged that both methods may suffer from systematic and random measurement error [62], which is a limitation. The objectively measured cardiometabolic risk markers are less prone to bias than subjective self-reports of dietary intake, and the changes in dietary fat intake could, to some extent, be reflected in objectively measured serum lipids.

A limitation of our study is that we could not blind the participants due to the study design, which is a weakness with lifestyle interventions in general. It is also a limitation that the person who performed the analyzes was not blinded to intervention allocation. In our study, only laboratory personnel were blinded. The fact that the groups were aware of their group allocation may have affected the results relating to dietary intake by increasing the risk of intentional misreporting due to for example social desirability. The intervention group may have reported better dietary habits in both dietary assessments to appear healthier given that the aim of the study was to evaluate dietary habits after a dietary mHealth intervention. However, our results of an increased NNR-score in the control group, and not in the intervention group, contradicts this. Additionally, unintentional misreporting, due to for example recall bias, may be present in FFQ assessments, but less so in the dietary recordings. Further, objectively measured anthropometrics are not affected by misreporting due to participants knowing their group belonging. Another limitation is the missing data from the dietary assessments, with lower response rates from the dietary record than from the FFQ, possibly due to the higher burden of keeping a food diary. Nevertheless, our results from analyses using data from the record and FFQ point in the same directions. In addition, it is a limitation to assess absolute intakes and changes in dietary intake with an FFQ.

It is a limitation that the control group was given the app at the 3-month follow-up, which meant that we could only address the intervention effect between baseline and 3 months. Further, the power calculation was not based on our primary outcome i.e. diet quality. The a priori power calculation was performed using HbA1c as no previous mHealth intervention has addressed adherence to the NNR. Finally, it is a limitation that we did not reach the number of participants suggested by our power calculation. This may have led our study to be underpowered for detecting changes in dietary intake or cardiometabolic risk markers. Our findings should therefore be considered hypothesis-generating rather than conclusive, highlighting the need for further studies to confirm our results.

## Conclusion

We found no effect of a smartphone-delivered dietary education on overall diet quality among people with type 2 diabetes. Our results indicated potential positive effects of the intervention on dietary fat intake and triglyceride levels, but these findings must be confirmed in future studies.

## Supplementary Information

Additional file 1. CONSORT checklist.

Additional file 2. Supplementary material including Table S1-S2.

## Data Availability

Participant data analyzed in the current study are not publicly available due to ethical reasons but are available from the principal investigator (SEB) upon reasonable request.

## Abbreviations

Apps: (Mobile) Applications
BMI: Body Mass Index
CI: Confidence interval
FFQ: Food Frequency Questionnaire
HAPPY: Healthy Eating using APP technologY
HbA1c: Glycated hemoglobin
HDL: High-density lipoprotein cholesterol
LDL: Low-density lipoprotein cholesterol
mHealth: Mobile health
NNR: Nordic Nutrition Recommendations
SD: Standard Deviations
