# Supplementary material for: The effect of an app-based dietary education on dietary intake and cardiometabolic risk markers in people with type 2 diabetes: results from a randomized controlled trial
Source: Nutr J. 2025 Jan 4;24:2. doi: 10.1186/s12937-024-01069-2 (PMC11699681; doi:10.1186/s12937-024-01069-2)
Supplement: Supplementary file 2 — Additional file 2. Supplementary material including Table S1-S2. [file 12937_2024_1069_MOESM2_ESM.docx]

**Supplementary material**

Sjöblom et al. The effect of an app-based dietary education on dietary intake and cardiometabolic risk markers in patients with type 2 diabetes: a randomized clinical trial.

| **Supplemental Table S1.** Food items included and adjustments for weight of composite dishes from the 4-day dietary record included in the HAPPY trial. | | | | | | |
| --- | --- | --- | --- | --- | --- | --- |
| **Food categories dietary record** | **Food items included** | **Food category in analysis** | **Notes** | **Comments** | **Adjustment or Assumption of composite dishes** | **Calculation for Adjustment of weights of composite dishes (if applicable)** |
| **Vegetables** | All reported vegetables | Fruit and vegetables | E.g., Dark green leafy vegetables, Avocado, Cauliflower, Celery, Broccoli, Onions, Corn, Squash, Fresh herbs, Ginger, Olives, Peppers, Fermented vegetables, Capers, Ginger | Includes fresh, frozen, canned and cooked vegetables. | Vegetables included from composite dishes have been calculated as a weight ratio. | Greek salad 80% salad 20% cheese/meat/other; Pasta salad 50% salad 50% pasta; White cabbage/cauliflower/spinach stew homemade 70% white cabbage/cauliflower/spinach 30% other; Eggplant puree 80% Eggplant 40% other; Tomato sun-dried in oil 70% tomato (drained weight); Cauliflower puree soup 50% cauliflower 50% other; Stew ratatouille veg 50%; Spinach/cauliflower soup veg. 20% spinach/cauliflower 80% cream/other: Vegetable mixture with soybeans 30% soybeans 70% vegetables. |
| **Fruit** | All reported fruits | Fruit and vegetables | E.g., Banana, Apple Pomegranate, Grapefruit, Melon, Pineapple, Kiwi, Citrus fruits, Physalis, Mango, Grapes, Apple/pear, Sharon, Rhubarb | Includes fresh, frozen, and canned fruits. | Fruit included from composite dishes have been calculated as a weight ratio. | Smoothie 50% fruit 50% yoghurt/other |
| **Berries** | All reported berries | Fruit and vegetables | E.g., Raspberry, Cloudberry, Strawberry, Blackberry, Blueberry, Cherry, Lingonberry, Cranberry, currant and Juniper | Includes fresh, frozen and dried berries. | No adjustment | No adjustment |
| **Root vegetables** | All reported root crops | Fruit and vegetables | E.g., Carrot, Parsnip, Pumpkin, Beetroot, Turnip, Celeriac, Horseradish | Includes fresh, frozen, canned and cooked root vegetables. | Root vegetables included from composite dishes have been calculated as a weight ratio. | Carrot soup 50% carrot/potato 50% water/other; Beetroot salad 80% beetroot 20% sour cream/other; Stir-fried vegetables Asian stir-fried with rapeseed oil 95% wok 5% oil; Peas carrot boiled 50% carrot 50% peas |
| **Legumes** | All reported legumes | Legumes/pulses | E.g., Soybeans, Black/Kidney Beans, Green Peas, Chickpeas, Red/Green Lentils, Broad Beans, Mung Beans, Edamame Beans | Includes fresh, dried, boiled, canned and cooked legumes. | Legumes included from composite dishes have been calculated as a weight ratio. | Beans/lentils/pea stews 30% Beans 70% other/water; White beans with. canned tomato sauce 40% beans 60% sauce; Peas carrot boiled 50% peas 50% carrot; Vegetable mixture with soybeans 30% soybeans 70% vegetables: Lentil soup/pea soup veg. 20% lentils/peas 80% water/other |
| **Lean fish** | All reported lean fish | Total fish and seafood | E.g., Cod, Tuna, Saithe, Pollock, Halibut, Zander | Includes fresh, frozen, canned, boiled, and fried lean fish | Lean fish included from composite dishes have been calculated as a weight ratio. | Tuna salad; 20% tuna 80% salad/other; Tuna Tomato & basil Abba 60% tuna & 40% other; Cod balls Broccoli & carrot ICA 60% cod 40% other |
| **Fatty fish** | All reported fatty fish | Total fish and seafood | E.g., Salmon, Herring, Char, Cod, Saithe, Baltic herring, Mackerel, Sardine | Includes fresh, frozen, canned, boiled, smoked, oven roasted and fried fatty fish | Fatty fish included from composite dishes have been calculated as a weight ratio. | Sushi nigiri with salmon 50% fish and 50% rice; Sushi maki with salmon 30% fish 70% rice; Canned sardines in oil 68% sardines 32% oil; Canned sardines or mackerel in tomato sauce 50% sardines/mackerel 50% tomato sauce; Salmon burger 60% salmon 40% bread/other |
| **Fish other** | All other/unspecified fish | Total fish and seafood | E.g., Fish balls, Minced fish, Breaded fish, Fish burger, Squid | Includes fresh, boiled, fried, smoked, oven roasted unspecific fish | Unspecific fish included from composite dishes have been calculated as a weight ratio. | Fish burger 60% salmon 40% bread/other; Fish balls 60% fish 40% Sause; Fried squid 95% squid 5% oil/other; Fish soup with root vegetables 10% fish 90% water/cream/other |
| **Seafood** | All reported seafood | Total fish and seafood | E.g., Shellfish, Shrimps, Crayfish, Oysters, Mussel, Crab, Lobster | Includes fresh, frozen, canned, cooked and fried | Seafood included from composite dishes have been calculated as a weight ratio. | Deep-fried shrimp prepared at a restaurant 95% shrimp 5% oil/other |
| **Red and processed meat** | All reported red and processed meat | Red and processed meat | E.g., Beef, Pork, Sausage, Lamb, Game meat | Includes fresh, raw, frozen, boiled, fried, smoked, oven-roasted, grilled | Red and processed meat included from composite dishes have been calculated as a weight ratio. | Stew 60% meat 40% sauce/other; Soup 10-30% meat 70-90% sauce/water/other; Minced meat sauce 60% meat 40% tomato sauce/other; Taco shell with minced meat 50% meat 50% taco shell/vegetables/other; Sausage stroganoff 60% sausage 40% sauce/other; Hamburger 60% (double 70%) meat 40% bread/other; Sausage with bread 70% sausage 30% bread; Beef spit 18% meat 82% potatoes/other; Stew Indian lamb stew 40% lamb & 60% sauce/other; Fully roasted cutlet Meal Frozen Familjen Dafgård 15% meat 85% sauce/other; Gooh Meatballs mashed potatoes 30% meat 70% sauce/other |
| **Sugar-sweetened beverages** | All reported sugar-sweetened beverages | Sugar-sweetened beverages | E.g. Coca-Cola, Fanta, Pepsi or juice with added sugar such as strawberry juice | Includes sugar-sweetened beverages such as soft drinks but also juices that have added sugar such as mixed juices with water | No adjustment | No adjustment |
| **Juice** | All reported fruit and vegetable juices | Sugar-sweetened beverages | E.g., Apple juice, Orange juice, Grapefruit juice, Beetroot juice | Includes freshly squeezed juices or store-bought juices, juices without added sugar | No adjustment | No adjustment |
| Abbreviations: HAPPY, Healthy Eating using APP technologY. | | | | | | |

| **Table S2.** Nordic Nutrition Recommendations 2023 (NNR) score based on the food-based dietary guidelines and cut-offs points for adherence. | | |
| --- | --- | --- |
| **NNR-score components** | **Recommended intake** | **Total NNR-score, 0-30 points (10 components, 0-3 points each)** |
| 1) Total vegetable, fruits and berries, g/day | ≥500-800 | 0 points: <100 g/day  1 point: 100-299 g/day  2 points: 300-499 g/day  **3 points: ≥500 g/day** |
| 2) Cereals, whole grains, g/day | ≥90 | 0 points: <30 g/day  1 point: 30-59 g/day  2 points: 60-89 g/day  **3 points: ≥90 g/day** |
| 3) Pulses/legumes, g/day | No specific cut-off^a^ | 0 points: <20 g/day  1 point: 20-49 g/day  2 points: 50-79 g/day  **3 points: ≥80 g/day** |
| 4) Nuts and seeds, g/day | 20-30 | 0 points: 0 g/day  1 point: 1-9 g/day  2 points: 10-19 g/day  **3 points: 20-30 g/day**  2 points: 31-40 g/day  1 point: 41-50 g/day  0 point: >50 g/day |
| 5) Total fish and seafood, g/week | 300-450 | 0 points: <100 g/week  1 point: 100-199 g/week  2 points: 200-299 g/week  **3 points: 300-450 g/week**  2: points: 451-550 g/week  1 point: 551-650 g/week  0 points: >650 g/week |
| 6) Red meat, g/week | ≤350 | 0 points: >500 g/week  1 point: 426-500 g/week  2 points: 351-425 g/week  **3 points: ≤350 g/week** |
| 7) Milk and dairy, g/day | 350-500 | 0 points: <150 g/day  1 point: 150-249 g/day  2 points: 250-349 g/day  **3 points: 350-500 g/day**  2 points: 501-599 g/day  1 point: 600-699 g/day  0 points: >700 g/day |
| 8) Vegetable oils, g/day | ≥25 | 0 points: <5 g/day  1 point: 5-15 g/day  2 points: 16-24 g/day  3 points: ≥25 g/day |
| 9) Sweets and confectioneries, incl. sugar-sweetened beverages, g/day | No specific cut-off^b^ | 0 points: >200 g/day  1 point: 150-199 g/day  2 points: 100-149 g/day  **3 points: <100 g/day** |
| 10) Alcohol, g/day | No safe limit | 0 points: >12 g/day  1 point: 7-12 g/day  2 points: 1-6 g/day  **3 points: 0 g/day** |
| 11) Potatoes | No specific cut-off | Not included |
| 12) While meat | No specific cut-off | Not included |
| 13) Eggs | No specific cut-off | Not included |
| ^a^Although it is recommended as a significant part of the Nordic diet to support nutrient and protein intake, legumes do not have a specified intake cut-off. On the basis of the Swedish Food Composition Database from the Swedish National Food Agency, a standard portion of legumes (beans and lentils) is approximately 75-100 grams per day [29]. Therefore, we defined the recommended intake as 80 grams of legumes per day, which was given 3 points in our score.  ^b^The guidelines for sweets, including chocolate, cakes, biscuits, confectioneries, and sugar-sweetened beverages, lacks a specified intake limit although their high energy and added sugar content warrants limited consumption [33]. The recommendation for added sugar is less than 10% of the total energy, corresponding to approximately 50-75 grams of free sugar per day for an adult [33]. Therefore, we defined the recommended level of sweets as less than 100 grams per day, which includes the total weight of foods containing added sugar. | | |
